# Supplementary material for: Design, Synthesis and Antiproliferative Evaluation of Bis-Indole Derivatives with a Phenyl Linker: Focus on Autophagy
Source: Molecules. 2022 Dec 28;28(1):251. doi: 10.3390/molecules28010251 (PMC9822133; doi:10.3390/molecules28010251)
Supplement: Supplementary file 1 [file molecules-28-00251-s001.zip › molecules-2105944-supplementary.pdf]

molecules

MDPI

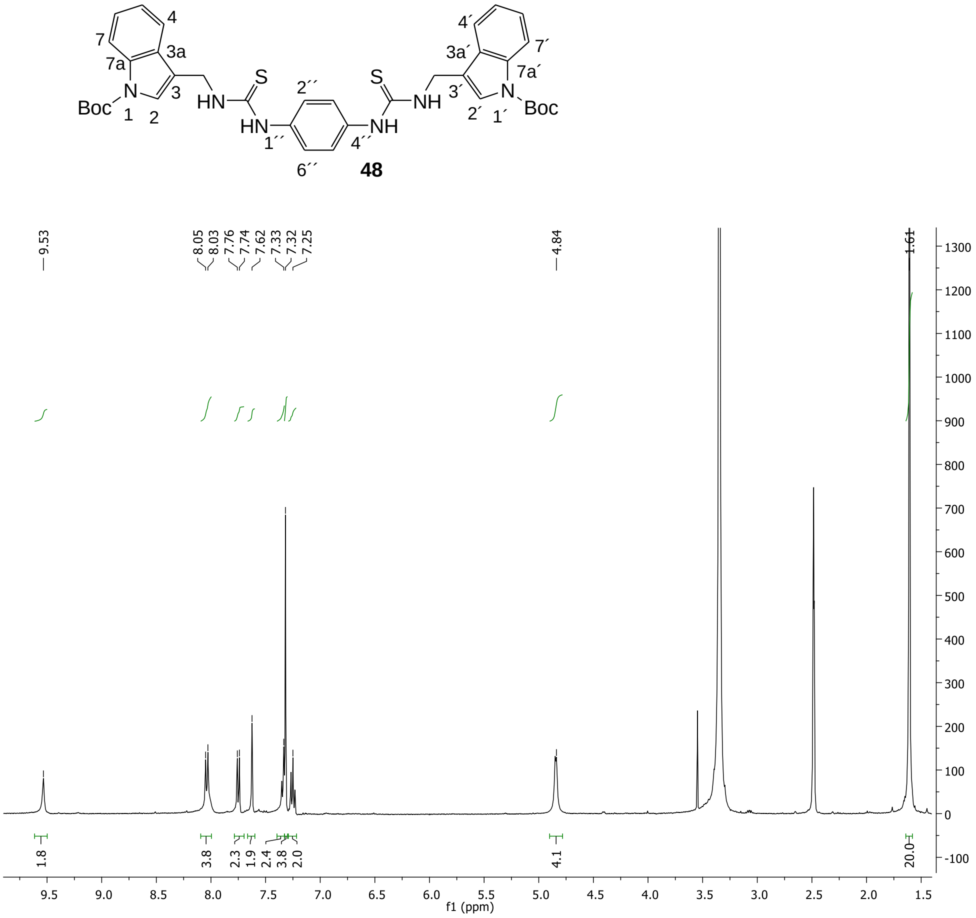

^{1}H NMR spectrum (DMSO-*d6*, 400 MHz) of thiourea **48**.

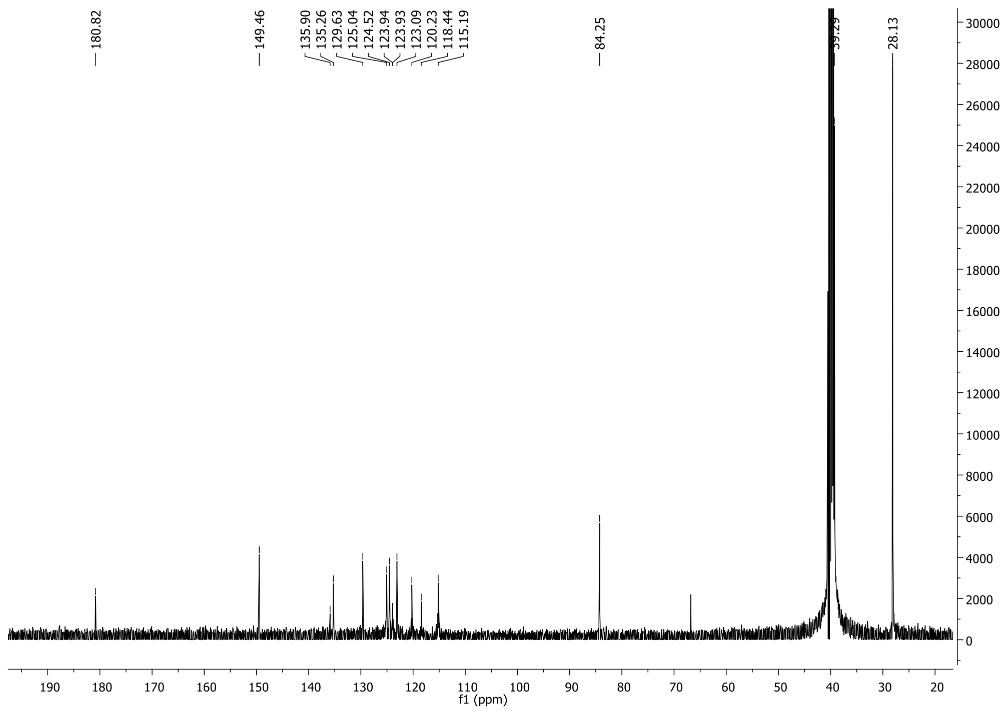

^{13}C NMR spectrum (DMSO-*d6*, 100 MHz) of thiourea **48**.

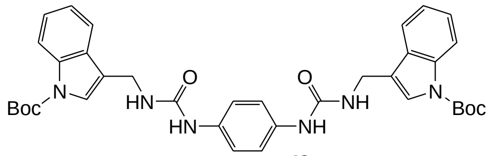

49

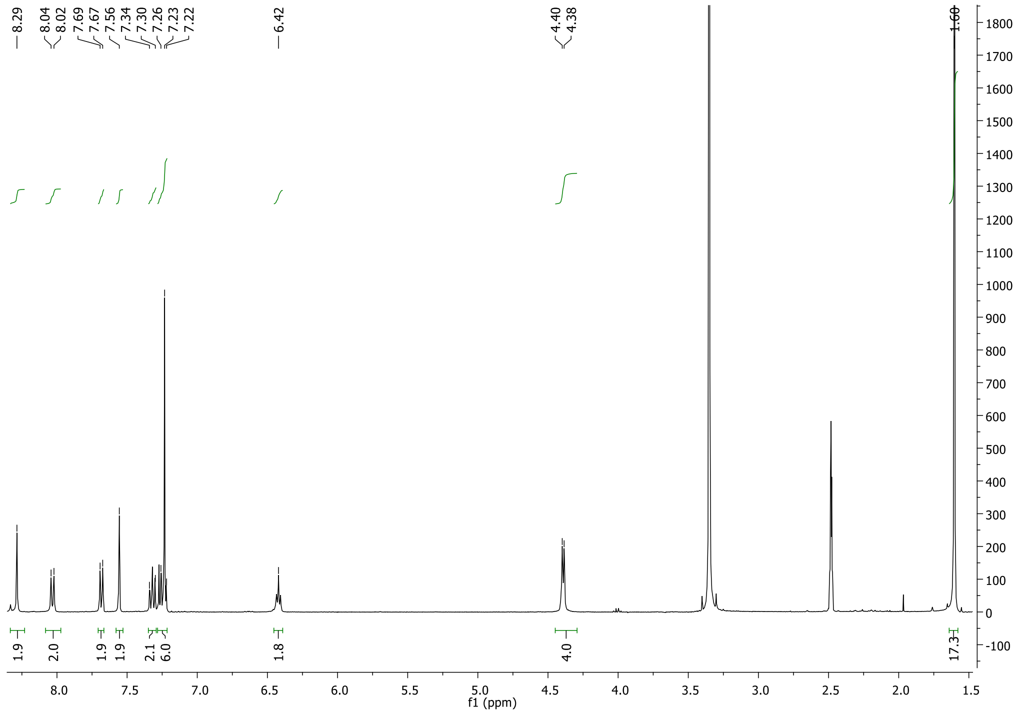

^{1}H NMR spectrum (DMSO-*d6*, 400 MHz) of urea **49**.

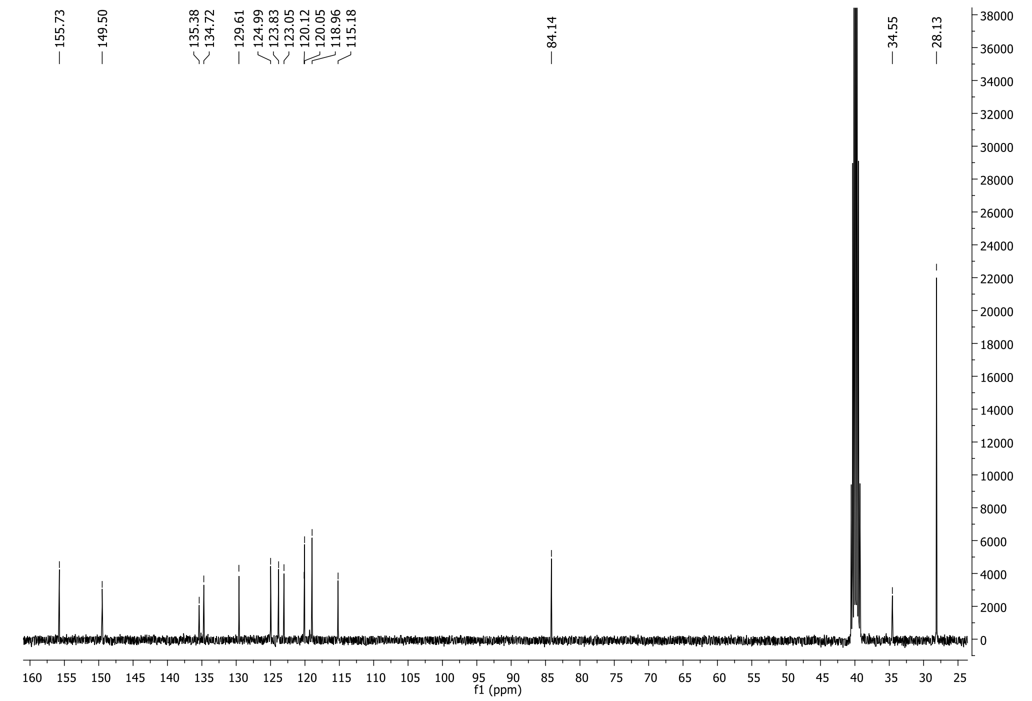

^{13}C NMR spectrum (DMSO-*d6*, 100 MHz) of urea **49**.

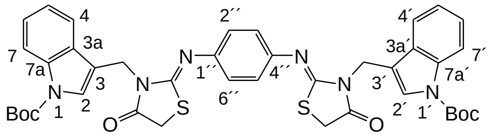

51a

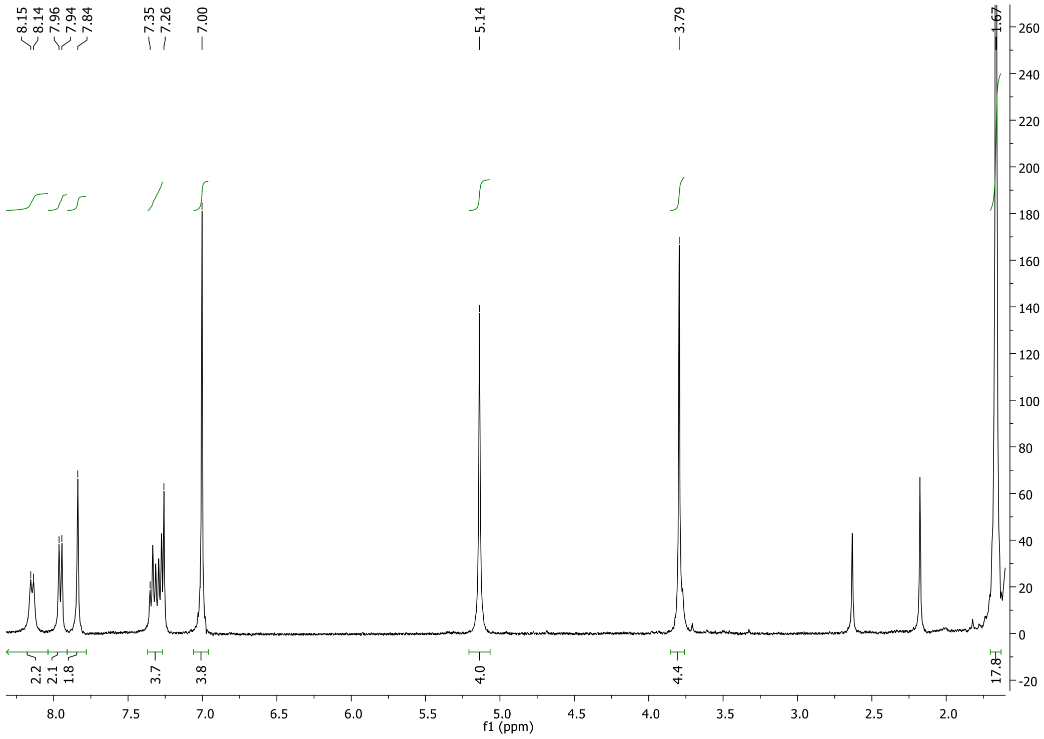

^{1}H NMR spectrum ($CDCl_3$, 400 MHz) of thiazolidin-4-one **51a**.

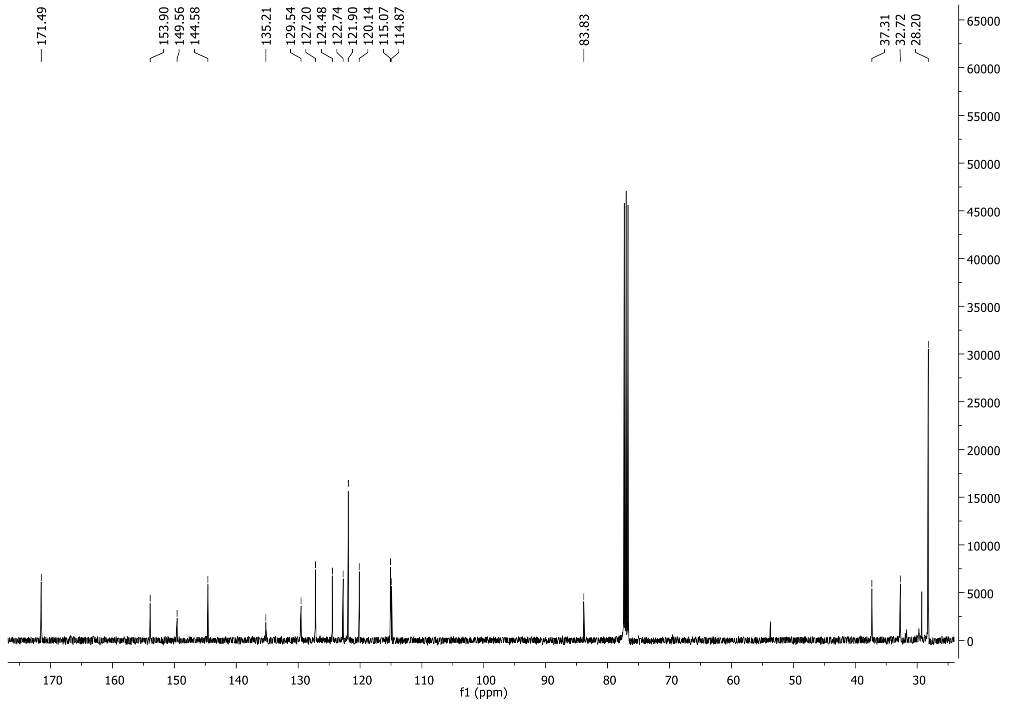

^{13}C NMR spectrum ($CDCl_3$, 100 MHz) of thiazolidin-4-one **51a**.

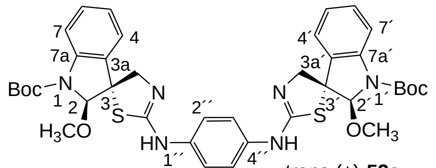

trans-(±)-**52a**

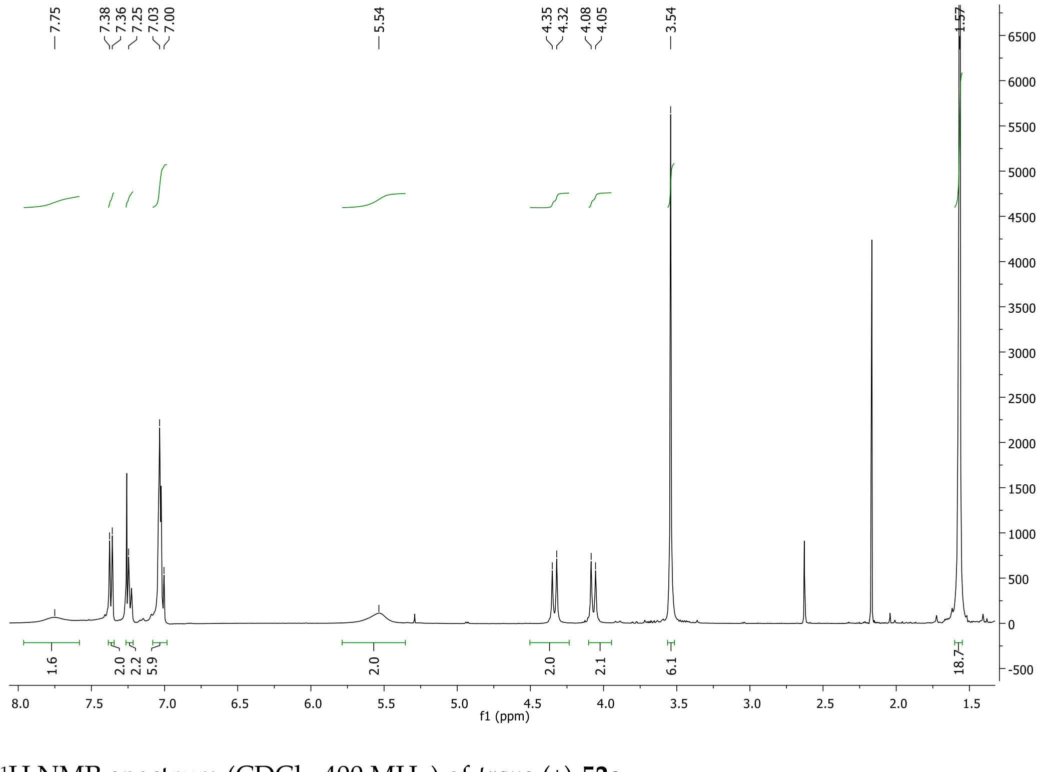

^{1}H NMR spectrum ($CDCl_3$, 400 MHz) of *trans*-(±)-**52a**.

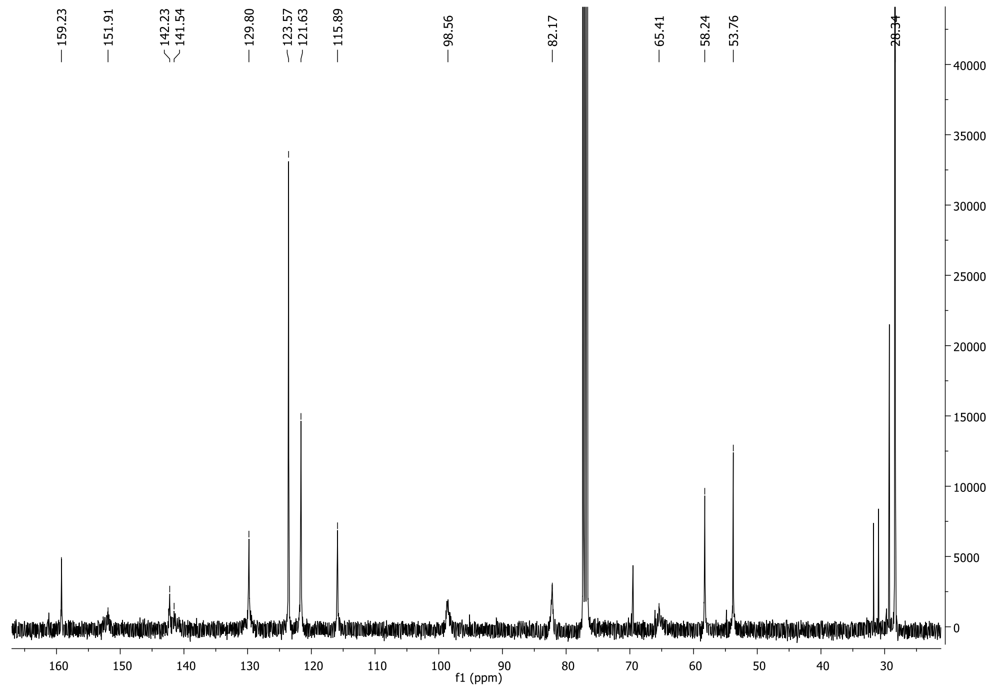

^{13}C NMR spectrum ($CDCl_3$, 100 MHz) of *trans*-(±)-**52a**.

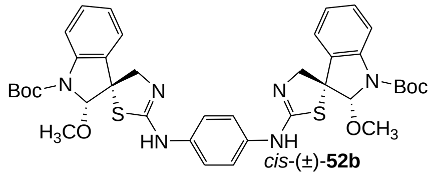

cis-(±)-**52b**

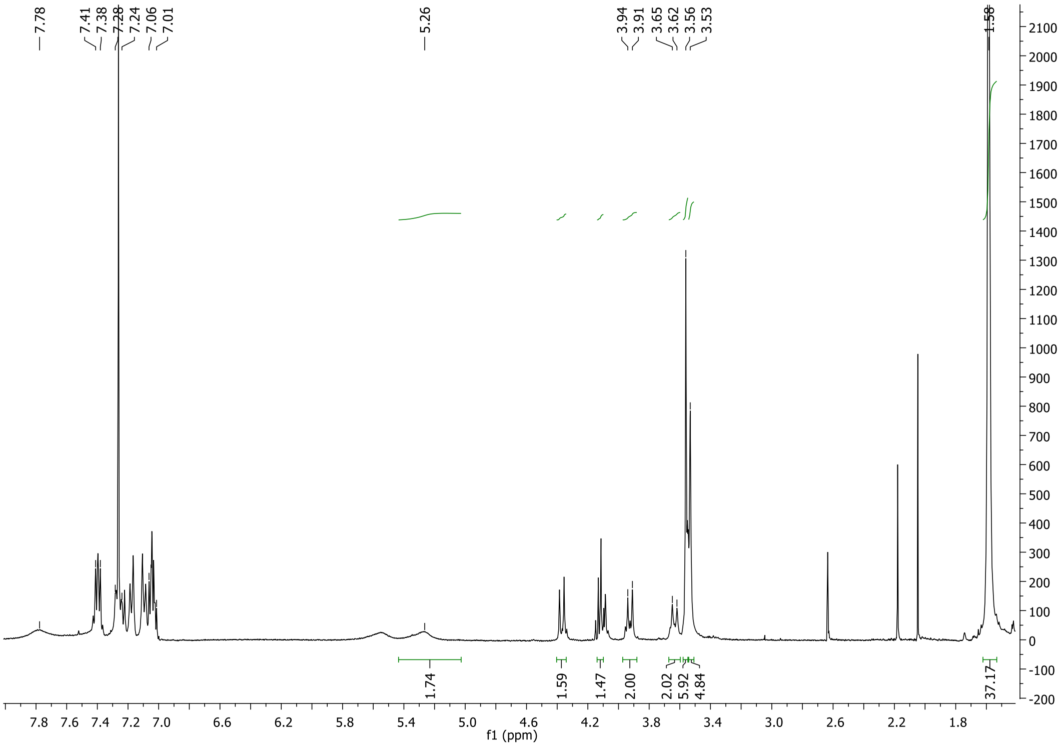

^{1}H NMR spectrum ($CDCl_3$, 400 MHz) of cis-(±)-**52a**.
